# Sequential organ failure assessment in predicting mortality after paraquat poisoning: A meta-analysis

**DOI:** 10.1371/journal.pone.0207725

**Published:** 2018-11-16

**Authors:** Wen Jie Wang, Li Wei Zhang, Shun Yi Feng, Jie Gao, Yong Li

**Affiliations:** 1 Emergency Department, Cangzhou Central Hospital, Yunhe Qu, Cangzhou, China; 2 Laboratory Department, Yutian County Hospital, Yutian Xian, Tangshan, China; The Ohio State University, UNITED STATES

## Abstract

Sequential organ failure assessment (SOFA) score is commonly used to determine disease severity and predict prognosis in critically ill patients. However, the prognostic value of SOFA after acute paraquat (PQ) poisoning remains unclear. This meta-analysis aimed to study the capability of SOFA to predict mortality in patients with PQ poisoning. Databases that included PubMed, Embase, Web of Science, ScienceDirect, Embase, and Cochrane Library were searched through May 2018. Six studies involving 946 patients were included in the meta-analysis. Study-specific odds ratios (ORs) and 95% confidence intervals (CIs) were calculated, and then ORs with 95% CIs were pooled for the estimation of the prognostic role of SOFA in patients with PQ poisoning. Results showed that higher SOFA in patients with PQ poisoning was related to severe mortality (OR = 8.14, 95%CI 4.26–15.58, p<0.001). The pooled sensitivity, specificity, positive likelihood ratio, negative likelihood ratio, diagnostic OR, and area under the curve were 72% (95%CI 0.65–0.79), 75% (95%CI 0.65–0.83), 2.9 (95%CI 2.0–4.1), 0.37 (95%CI 0.28–0.41), 8 (95%CI 4–14), and 0.79 (95%CI 0.76–0.83), respectively. No evidence of publication bias was detected by funnel plot analysis and formal statistical tests. Sensitivity analyses showed no important differences in the estimates of effects. The high SOFA score (8.1-fold) was associated with severe mortality in patients with PQ poisoning.

## Introduction

As a highly effective, nonselecting, and fast-acting herbicide, paraquat (PQ) is harmless to the environment because of its rapid decomposition into nontoxic compounds after soil contact. These excellent properties led to the adoption of PQ worldwide over the past few decades. PQ ingestion occurs frequently in the agricultural countryside, either accidentally or as a suicide attempt, thereby posing a serious public health problem. PQ accounts for an estimated 20 deaths per million person-years worldwide [1–3]. Although many investigators have attempted to find efficacious treatments for the management of acute PQ poisoning, the clinical therapeutics are still unsatisfactory, and the mortality rate remains tremendously high (50%–90%) [2, 4, 5]. Therefore, confirming the diagnosis of PQ poisoning and risk assessment in a timely manner are particularly important.

Toxicological analysis of plasma and urine samples is used to establish the diagnosis. The mortality rate of PQ poisoning is directly related to plasma and urine PQ concentrations. Unfortunately, PQ assays are not widely available, particularly in developing countries. Another important predictor of mortality is the amount of PQ consumed [6]. However, estimates on the amount ingested are often unobtainable or unreliable in many intoxicated patients. Current studies propose some predictive equations based on similar sample sizes. Unfortunately, these equations have not been widely used in clinical situations because of their difficult calculation [7, 8]. Therefore, alternative prognostic indicators for acute PQ poisoning are still required for clinical practice.

Sequential organ failure assessment (SOFA) score calculates a summary value for the degree of dysfunction of six parameters (PaO_2_/FiO_2_, platelet count, serum bilirubin level, hypotension, Glasgow coma score, and serum creatinine or urine output). Four levels of dysfunction are identified in the SOFA score for each organ system. This scoring system has been commonly used to assess the severity and prognosis of diseases, especially in intensive care units. Currently, SOFA can predict the prognosis and mortality of patients with PQ poisoning [9–14]. However, these studies[9–14] showed a discordant predictive value of this scoring system. Therefore, the present study aimed to investigate the potential role of SOFA as a prognostic marker in patients with PQ poisoning.

## Materials and methods

This meta-analysis was performed in accordance with the guidelines of Preferred Reporting Items for Systematic Reviews and Meta-Analyses (PRISMA) guidelines [15], and it was conducted following a priori established protocol (PROSPERO: CRD42018095915). Ethical approval was not required because this study was a literature-based work.

### Search strategy

The related clinical research was obtained from the electronic databases, including PubMed, Web of Science, ScienceDirect, Embase, and Cochrane Library, using terms such as paraquat, predictive, prediction, prognostic, and sequential organ failure assessment, and a deadline of May 1, 2018. Simultaneously, references in corresponding literature included in the above databases were retrieved artificially based on the title of the literature to screen applicable studies.

### Inclusion and exclusion criteria

Inclusion criteria were as follows: focus on the association of SOFA with mortality risk in patients with PQ poisoning; the standard diagnostic criteria of PQ poisoning are met; and SOFA data are available to calculate odds ratio (OR) and its 95% confidence interval (CI). In cases of repeated studies or overlapping data, studies that involved large sample sizes or were published more recently were selected. If the reported data were incomplete, then the corresponding author was contacted to obtain complete data. Exclusion criteria were as follows: reviews, comments, abstracts, and case reports; literature with unavailable full text or data; and repeated publication.

### Data extraction

The substantial contents of each selected article were extracted by WJW and LWZ. Extracted information included the name of the first author, publication time, study design, sample size, mortality percentage, cut-off value, SOFA score, blood PQ level, time from ingestion to arrival and treatment protocol. Any disagreements were resolved by consensus or by consulting a third author (YL).

### Risk of bias

The risk of bias was independently evaluated by two reviewers (WJW and SYF) for each study as low, moderate, or high, using criteria adopted from the Quality Assessment of Diagnostic Accuracy Studies 2 (QUADAS-2) [16]. Conflict was resolved by discussion or by consulting a third author (YL).

### Statistical analysis

All pooled analyses were conducted using STATA 12.0 software (StataCorp LP, College Station, TX, USA). The Cochrane’s Q statistic and *I*^2^ statistic were computed to test the significance of potential heterogeneity. If studies reported moderate or low heterogeneity (*I*^2^ <50%), then the fixed effects model was used for pooling. Otherwise, the random effects model was adopted for *I*^2^ ≥50% [17]. The *I*^2^ statistic was used to evaluate heterogeneity; values of 25%, 50%, and 75% were considered low, moderate, and high heterogeneity, respectively [17]. Presence of heterogeneity warrant examining their sources where we used covariates in a meta-regression analysis. In this analysis, the following covariates were used: patients’ mortality percentage (≥50% vs. <50%), study design (prospective vs. retrospective), sample size (≥150 vs. <150), and cut-off value (≥3 vs. <3). We further conducted a sensitivity analysis to examine the impact of each study to the pooled effect. Begg’s test and Egger’s test were applied to assess publication bias among the included studies. Quality assessment of the included studies was conducted using RevMan 5.3 software (Nordic Cochrane Centre, Copenhagen, Denmark). p<0.05 was considered statistically significant.

## Result

### Literature search

**Fig 1:**
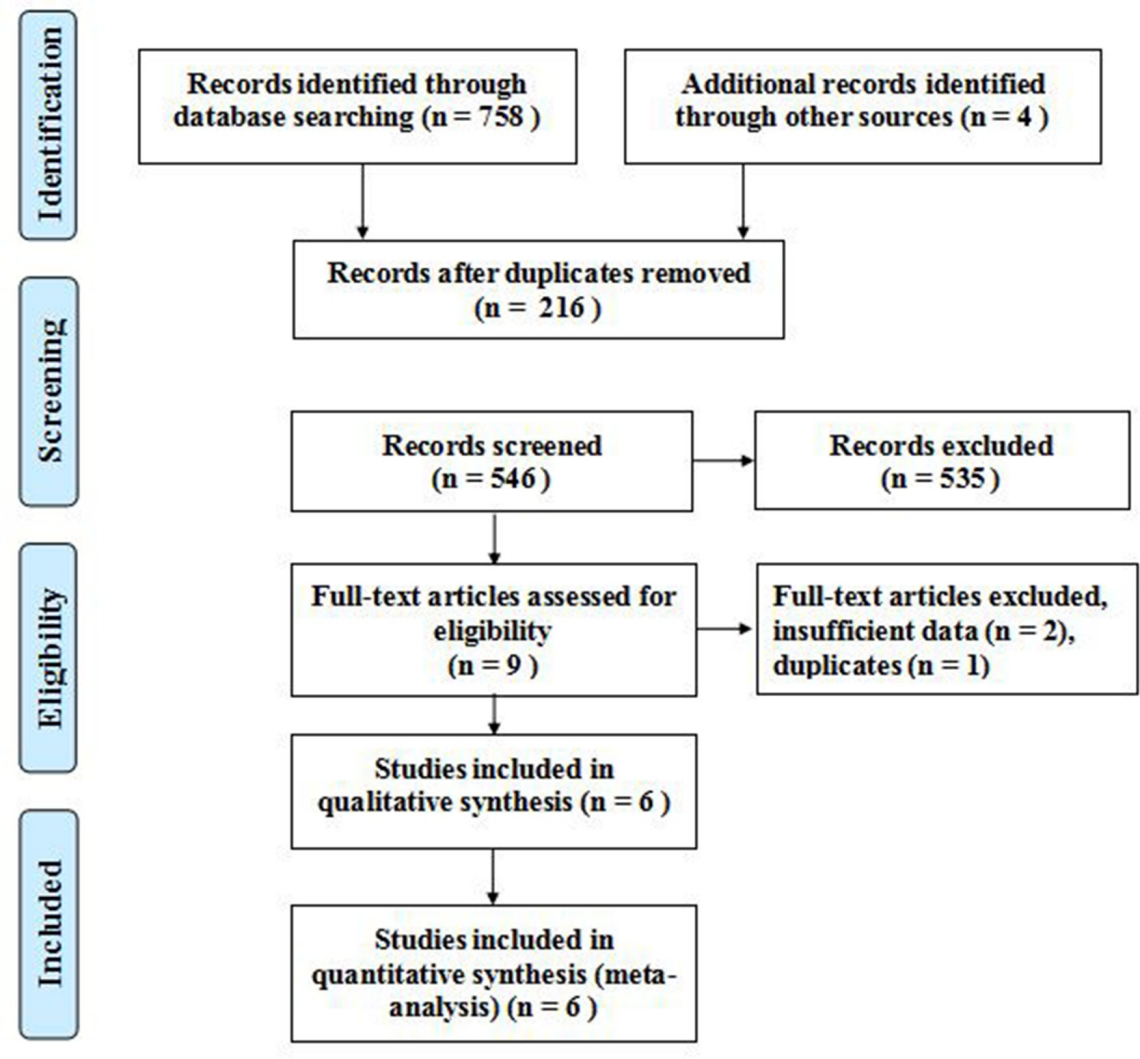
Risk of bias and applicability concerns.

On the basis of the search strategy, a total of 762 potentially relevant articles were identified in PubMed, Web of Science, ScienceDirect, Embase, and Cochrane Library. After browsing the titles and abstracts and then assessing the full text, six studies [9–14]. which included a total of 946 enrolled patients, were available for this meta-analysis. As shown in Fig 1, the literature search process is summarized in a flow diagram in accordance with PRISMA.

### Characteristics of the included studies

The baseline characteristics of these studies are listed in Table 1. The studies, which were published between 2012 and 2016, contained a total of 946 patients with a mean patient sample size of 157.67 (range 97–219). Of the six studies, five originated from China, and the remaining one originated from Korea.

**Table 1:** Characteristics of included studies.

First author	Publication year	Study Design	Sample size	Mortality (%)	Cut-off value	SOFA*		Blood PQ level (mg/L) *		Time from ingestion to arrival (h)		Treatment protocol
						Survivor	Non-survivor	Survivor	Non-survivor	Survivor	Non-survivor	
**Jiao et al**	2015	Retrospective	118	45.76	3	2.21±0.76	3.31±1.06	NA	NA	NA	NA	GL, HP, MP, CP, glutathione
**Kang et al**	2015	Prospective	97	42.27	2.5	2 (0–4)	5 (3–7)	0.1 (0.0–0.2)	0.8 (0.1–6.7)	14.3±5.5	14.1±5.9	GL, HP,MP, glutathione, vitamin C
**Lee et al.**	2016	Retrospective	219	80.37	3	1.6±1.9	3.5±2.7	NA	NA	NA	NA	GL, HP
**Li et al**	2015	Prospective	177	37.85	9	NA	NA	NA	NA	NA	NA	GL, HP, MP, CP
**Sun et al**	2016	Prospective	148	70.95	0.5	0.98±0.72	1.34±1.29	0.91±0.38	2.28±1.52	4.28±2.44	7.86±3.02	GL, HP, MP, glutathione, vitamin C
**Weng et al**	2012	Retrospective	187	54.01	3	2±2	4±2	1.4±2.0	7.6±6.1	19.1±26.9	8.7±12.9	GL, HP, MP, CP

NA = not available. SOFA = sequential organ failure assessment; PQ = paraquat; GL = gastric lavage HP = hemoperfusion; MP = methylprednisolone; CP = cyclophosphamide

*Continuous variable is presented as means ± SD or median (interquartile range) and categorical variable is presented as no. (%).

### Quality assessment

**Fig 2:**
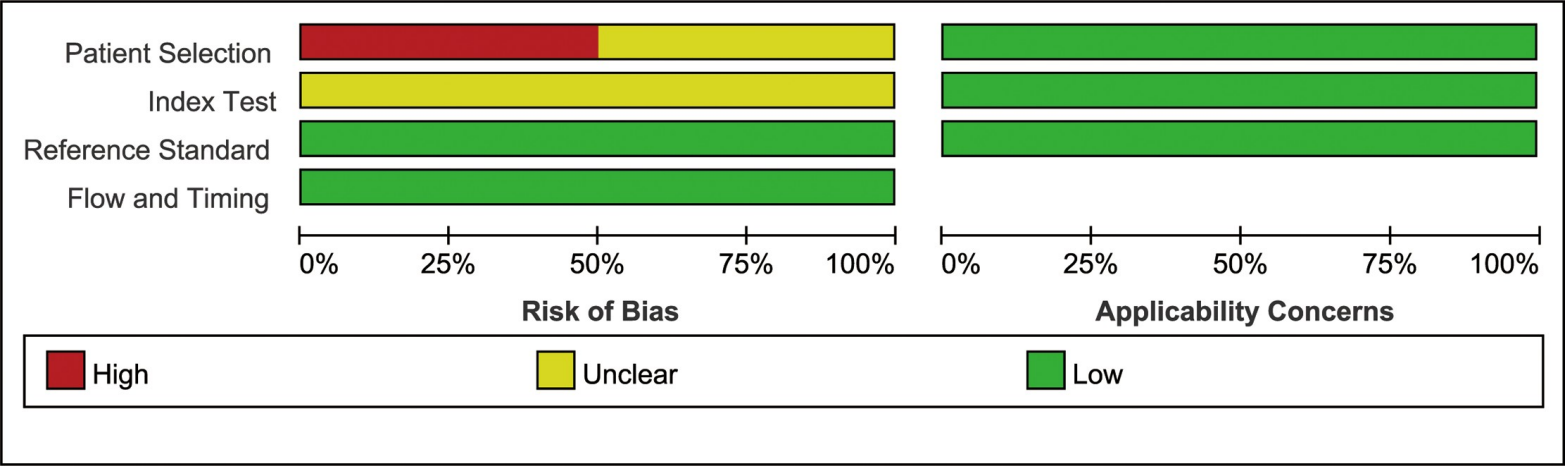
Forest plot for the association of SOFA and mortality.

According to the QUADAS-2, each of the six eligible studies included in our meta-analysis was assessed for quality. All of them demonstrated moderate to high quality, so they were appropriate for this meta-analysis (Fig 2).

### Meta-analysis of mortality

**Fig 3:**
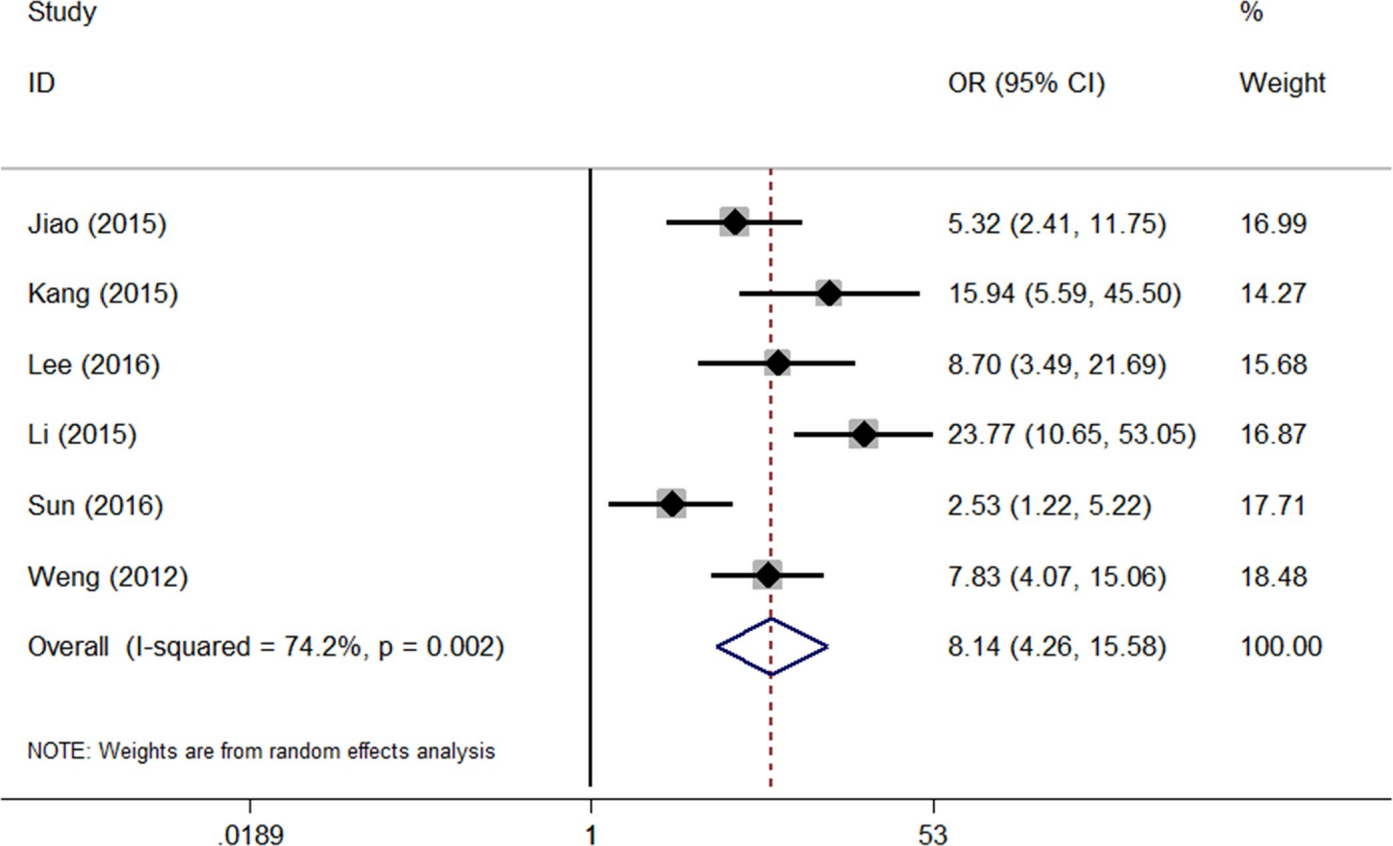
Risk of bias and applicability concerns.

**Fig 4:**
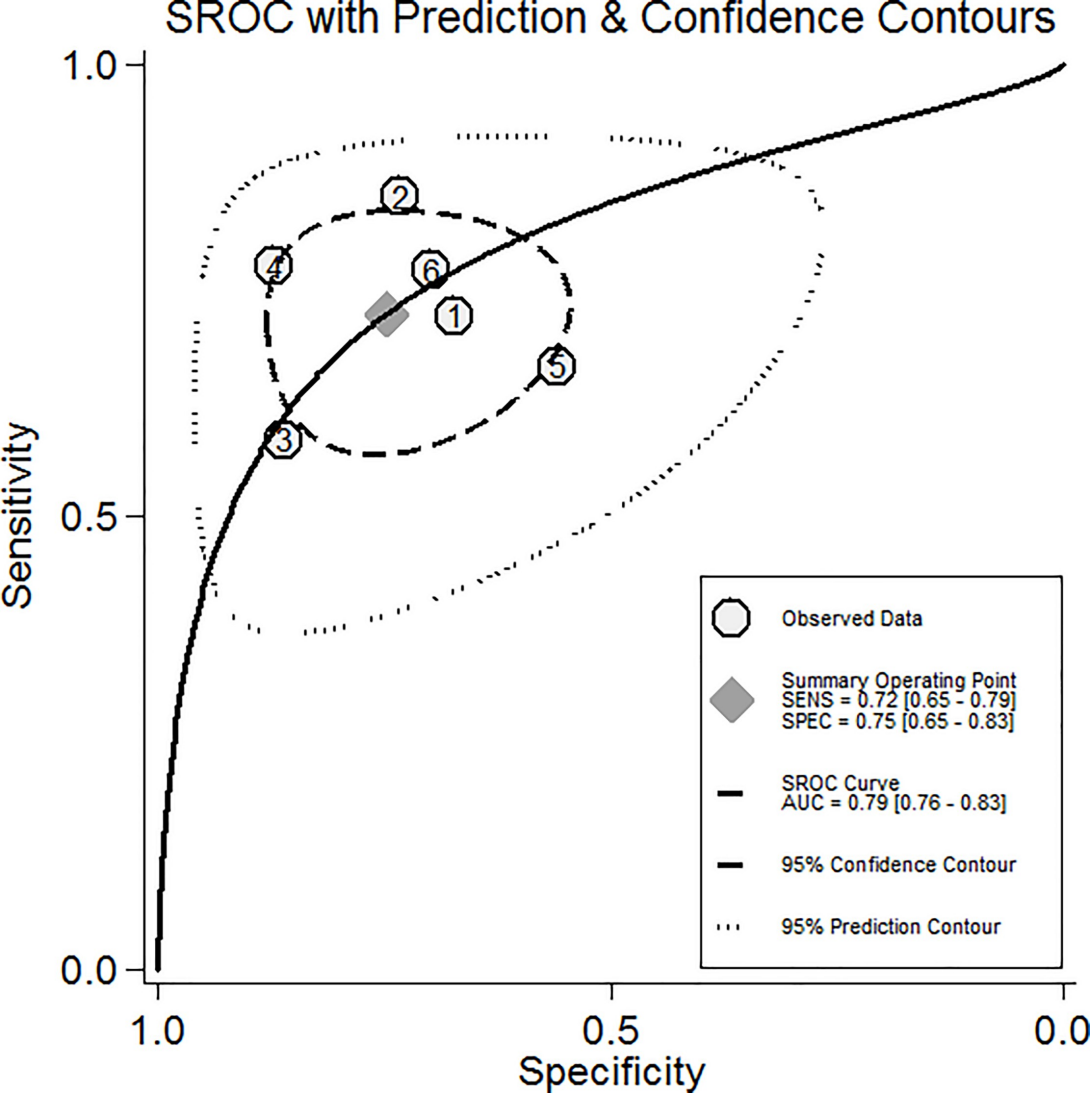
Summary ROC curve for the 6 included studies. Numbers in brackets are 95% CIs. AUC = area under ROC curve, SENS = sensitivity, SPEC = specificity.

The heterogeneity of the six studies was statistically significant (*I*^2^ = 74.2%, p<0.001). The random effects model was used to calculate the pooled OR and its 95% CI, yielding a significant difference (OR = 8.14, 95%CI 4.26–15.58, p<0.001). Thus, high SOFA in patients with PQ poisoning was related to severe mortality (Fig 3). The pooled sensitivity, specificity, positive likelihood ratio, negative likelihood ratio, and diagnostic OR were 72% (95%CI 0.65–0.79), 75% (95%CI 0.65–0.83), 2.9 (95%CI 2.0–4.1), 0.37 (95% CI 0.28–0.41), and 8 (95%CI 4–14), respectively. An area under the curve of 0.79 (95%CI 0.76–0.83) could effectively detect prognosis (Fig 4).

### Heterogeneity, sensitivity analysis, and publication bias assessment

**Fig 5:**
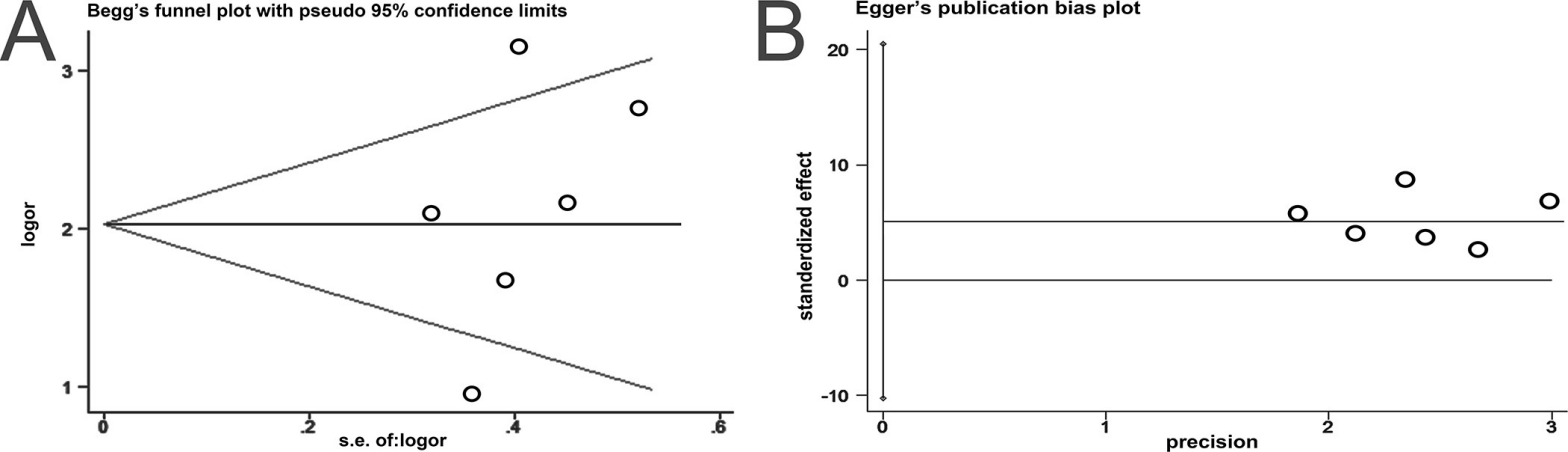
Funnel plot of the publication bias test. (A)Begg’s test; and (B) Egger’s test.

**Fig 6:**
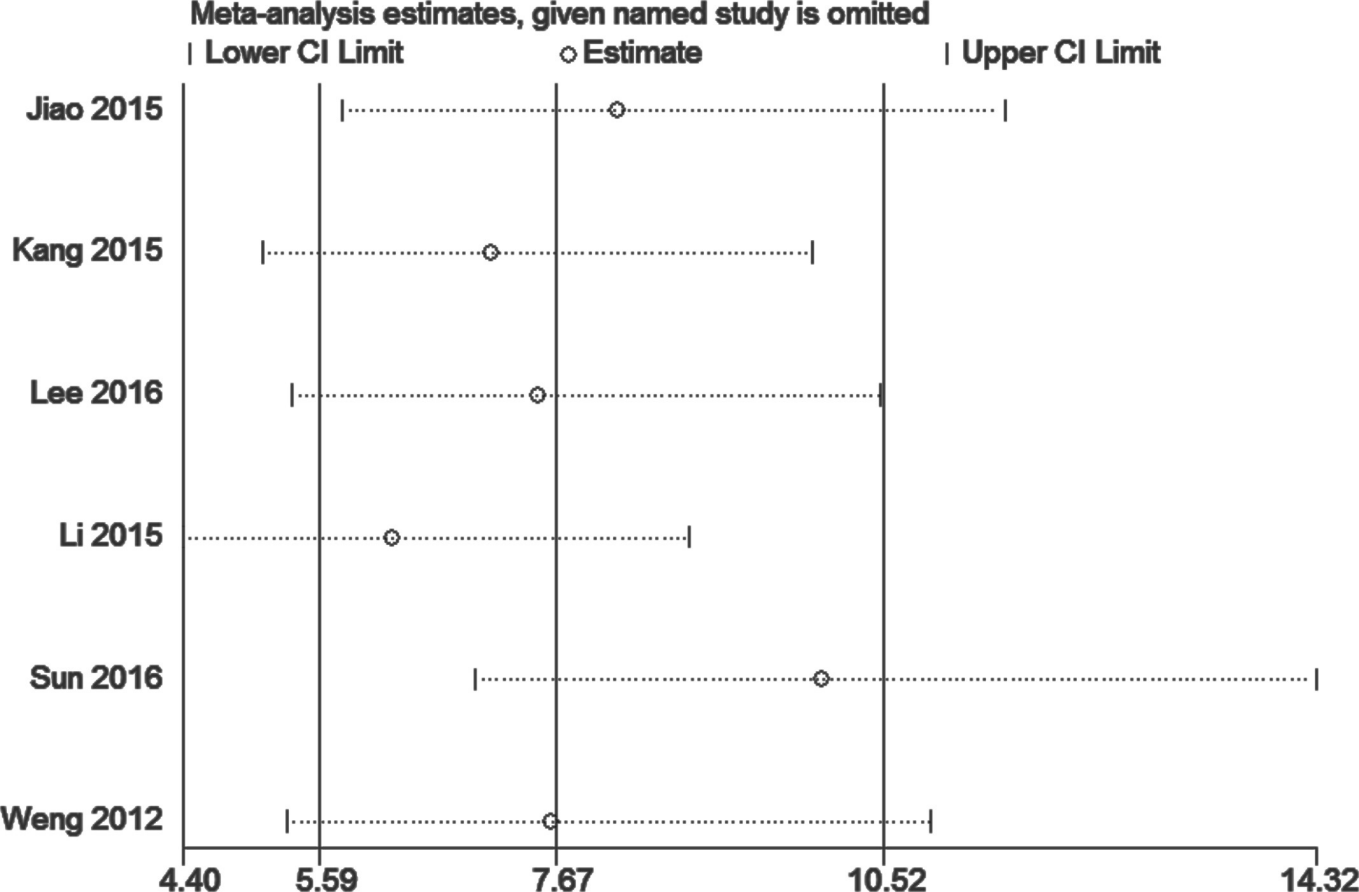
Sensitivity analysis of the relationship between SOFA and mortality.

Meta-regression analyses were performed in accordance with some covariates, including study design, sample size, mortality percentage, and cut-off value; however, meta-regression outcomes did not identify the sources of heterogeneity (Table 2). A funnel plot was employed to explore bias among the included studies. No apparent publication bias was detected (Begg’s test p = 0.260, Fig 5A; Egger’s test p = 0.406, Fig 5B). Given that no single study influenced the pooled effect, our result was considered robust (Fig 6).

**Table 2:** Meta-regression analysis of potential sources of heterogeneity.

Heterogeneity factors	Coefficient	SE	Z	P-value	95% CI (lower limit, upper limit)
**Design**	0.767	0.815	0.94	0.519	−9.593, 11.128
**Sample size**	2.264	0.813	2.79	0.219	−8.061, 12.588
**Mortality percentage**	1.842	0.651	2.83	0.216	−6.426, 10.111
**Cut-off value**	−1.864	1.299	−1.44	0.387	−18.367, 14.639

CI = confidence intervals, SE = standard error.

## Discussion

This study aimed to develop a reliable indicator to predict survival after PQ poisoning. Identifying the factors associated with early mortality may offer primary clinical information, which will be used to make correct evaluations and decisions and increase the chances of survival. Currently, the plasma PQ concentration is the most remarkable and consistent potential prognostic marker with acceptable sensitivity and specificity that can predict mortality [18–20]. We reviewed some articles [10, 11, 21] to validate and compare the performance of plasma PQ concentration and SOFA in various populations. The area under the ROC curves for plasma PQ concentration ranged from 0.679 to 0.866, and those for SOFA ranged from 0.631 to 0.867 in various populations.

Several variables inherent to SOFA, including low PaO_2_/FiO_2_, increased serum creatinine, elevated bilirubin levels, deteriorated state of circulation, inferior Glasgow coma score, and decreased platelet count, were associated with high mortality. Within the first few hours of PQ intoxication, PQ cation radicals with high affinity for alveoles directly damage the lungs and frequently cause death from hypoxemia and respiratory failure. Previous studies have shown that non-survivors have lower PaO2/FiO2 levels than survivors [9, 22]. Notably, a low PaO2/FiO2 level indicates a rapid and often fatal clinical evolution [23, 24]. As the kidney is the primary excretory pathway for absorbed PQ, PQ specially causes kidney damage. Renal failure may be manifested by proteinuria and oliguria, which then progresses to acute tubular necrosis [25]. Main lesions, including swelling, degeneration, and partial necrosis of epithelial cells, interstitial congestion, and edema, are located in renal proximal tubule [26]. Renal failure develops quite rapidly from moderate to severe PQ poisoning, and increased creatinine concentrations can be monitored for the detection of renal failure and prediction of long-term outcome [2, 9, 22, 27, 28]. The unchanged form of PQ are usually excreted in the urine, and, to a limited extent, in the liver and bile [29]. The liver is the main site for xenobiotic metabolism and has a high potential for generating reactive oxygen species. Thus, the liver is at high risk of suffering toxic damage [30]. Remarkable increase in bilirubin, alanine transaminase, and aspartate aminotransferase was observed in PQ-exposed patients [31–34]. Deteriorated circulation [35, 36] is also common in moderate and severe PQ poisoning. Initially, hypotension is generally due to hypovolemia, and then deteriorated lactic acidosis, hypoxemia, metabolic acidosis, and myocardial contraction asthenia generate circulatory failure [37]. PQ-intoxicated patients generally maintain a normal level of consciousness. Severe toxicity due to hypoxia, hypotension, and severe acidosis results in altered consciousness [38]. Toxic thrombocytopenia [22] was observed; however, the biological explanation for this clinical observation remains unclear. The causes of thrombocytopenia are multifactorial, including hypovolemia, hypothermia, circulatory failure, septicemia, and direct toxicity related to redox cycling. SOFA is based on the extent of organ function, and PQ poisoning is characterized by multi-organ failure; therefore, the SOFA system may be useful in predicting the prognosis of patients with acute PQ poisoning.

Given that significant heterogeneity was present in the evaluation of SOFA accuracy, our study explored factors that may be responsible for heterogeneity via meta-regression analysis. Although the specific covariates of patients and studies were examined, no factors could affect SOFA accuracy. The cause for this finding has yet to be determined.

To the best of our knowledge, we are the first to investigate the capability of SOFA through meta-analysis and to predict mortality in patients with PQ poisoning through a comprehensive literature search and the retrieval of all relevant trials. We used aggregated study-level data rather than individual patient data and thus we were able to use information from nearly all available trials. This approach enabled us render the results generalizable across a broad spectrum of patients with PQ poisoning. However, this meta-analysis study has some limitations that require the cautious interpretation of results. First, only six studies were included in this meta-analysis, which may inefficiently assess the accuracy of SOFA. Second, unpublished studies were not identified in our study, and no attempt was made to include articles in other languages. Third, significant heterogeneity was observed across trials. Four, the wide confidence intervals (OR = 8.14, 95%CI 4.26–15.58, p<0.001) may diminish the surety of any conclusions regarding efficacy [39]. Finally, the cut-off value varied among studies. In the perspective of statistical significance, this might introduce heterogeneity and bias to our pooled analysis. In addition, it made our meta-analysis fail to provide a precision guidance to clinical practice. Thus, in future more well-designed studies with large sample size are needed to solve this problem.

In conclusion, this meta-analysis of currently available studies proved that SOFA was likely an independent prognostic predictor for patients with PQ poisoning. Thus, clinicians should consider SOFA levels. However, further clinical trials with standardized methodology and criteria are required for confirmation.

## Supporting information

S1 PRISMA Checklist. (DOCX)

S2 PRISMA Flow diagram. (DOCX)
