# Supplementary material for: Sequential organ failure assessment in predicting mortality after paraquat poisoning: A meta-analysis
Source: PLoS One. 2018 Nov 16;13(11):e0207725. doi: 10.1371/journal.pone.0207725 (PMC6239328; doi:10.1371/journal.pone.0207725)
Supplement: S2 PRISMA — (DOCX) [file pone.0207725.s002.docx]

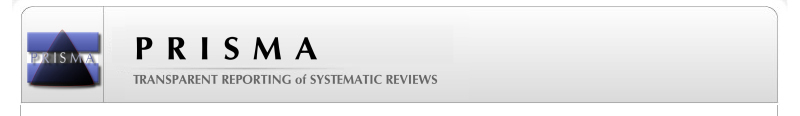
**PRISMA 2009 Flow Diagram**

**Records after** **duplicates removed
(n = 216 )**

**Records screened
(n = 546 )**

**Studies included in quantitative synthesis (meta-analysis) (n = 6 )**

**Full-text articles assessed for eligibility
(n = 9 )**

**Studies included in qualitative synthesis (n = 6 )**

**Full-text articles excluded,**

**insufficient data (n = 2),**

**duplicates (n = 1)**

**Records excluded
(n = 535 )**

## Included

## Eligibility

## Screening

## Identification

**Additional records identified through other sources (n = 4 )**

**Records identified through database searching (n = 758 )**
